# Interpretation of response categories in patient-reported rating scales: a controlled study among people with Parkinson's disease

**DOI:** 10.1186/1477-7525-8-61

**Published:** 2010-06-24

**Authors:** Ida Knutsson, Helena Rydström, Jan Reimer, Per Nyberg, Peter Hagell

**Affiliations:** 1 Department of Health Sciences, Lund University, PO Box 157, SE-221 00 Lund, Sweden; 2 Department of Neurology, Lund University Hospital, SE-221 85 Lund, Sweden

## Abstract

**Background:**

Unambiguous interpretation of ordered rating scale response categories requires distinct meanings of category labels. Also, summation of item responses into total scores assumes equal intervals between categories. While studies have identified problems with rating scale response category functioning there is a paucity of empirical studies regarding how respondents interpret response categories. We investigated the interpretation of commonly used rating scale response categories and attempted to identify distinct and roughly equally spaced response categories for patient-reported rating scales in Parkinson's disease (PD) and age-matched control subjects.

**Methods:**

Twenty-one rating scale response categories representing frequency, intensity and level of agreement were presented in random order to 51 people with PD (36 men; mean age, 66 years) and 36 age-matched controls (14 men; mean age, 66). Respondents indicated their interpretation of each category on 100-mm visual analog scales (VAS) anchored by *Never *- *Always*, *Not at all *- *Extremely*, and *Totally disagree *- *Completely agree*. VAS values were compared between groups, and response categories with mean values and non-overlapping 95% CIs corresponding to equally spaced locations on the VAS line were sought to identify the best options for three-, four-, five-, and six-category scales.

**Results:**

VAS values did not differ between the PD and control samples (P = 0.286) or according to educational level (P = 0.220), age (P = 0.220), self-reported physical functioning (P = 0.501) and mental health (P = 0.238), or (for the PD sample) PD duration (P = 0.213) or presence of dyskinesias (P = 0.212). Attempts to identify roughly equally spaced response categories for three-, four-, five-, and six-category scales were unsuccessful, as the 95% CIs of one or several of the identified response categories failed to include the criterion values for equal distances.

**Conclusions:**

This study offers an evidence base for selecting more interpretable patient-reported rating scale response categories. However, problems associated with raw rating scale data, primarily related to their ordinal structure also became apparent. This argues for the application of methodologies such as Rasch measurement. Rating scale response categories need to be treated with rigour in the construction and analysis of rating scales.

## Background

Patient-reported rating scales are gaining increasing importance in determining patient status and effectiveness of therapies. In such scales, responses to a number of items are typically summed to yield a total score intended to locate the respondent on a continuum from less to more on the variable of interest. Following the tradition of Likert [1], this is achieved by assigning integral numerals (e.g., 0 - 1 - 2 - 3) to descriptive response categories (e.g., none - mild - moderate - severe) as a means of partitioning the underlying latent quantitative continuum into successively increasing (or decreasing) amounts of the variable.

Although the summed rating scale approach may appear simple and straight forward, its appropriateness and legitimacy rests on some fundamental assumptions that often appear overlooked. First, for respondents to be able to communicate their positions accurately (and for investigators and clinicians to accurately interpret those responses), the descriptive response category labels need to have distinct and unambiguous meanings that reflect differences in amount [2]. Second, for arithmetic operations, such as summation of integral numerals assigned to response categories to be performed and interpreted legitimately, the magnitudes that successive categories represent need to be equally spaced [3,4]. Recently, these criteria have been emphasized by the U.S. Food and Drug Administration (FDA) for patient-reported rating scales to be considered appropriate as clinical trial endpoints [5].

Although attention has been paid to these and related issues in the behavioral and social sciences [2,6-8], less work appears to have been conducted in the clinical health sciences [9-11]. Furthermore, a considerable number of participants in available studies in the health arena have not suffered from any specific medical conditions [9-11]. Particularly, there seems to be a lack of this type of study in the clinical neurosciences. However, studies have shown that rating scale response categories often do not function as expected and required among people with neurological disorders such as Parkinson's disease (PD), multiple sclerosis and stroke [12-18]. These studies illustrate that although a larger number of response categories generally tend to increase variance and, hence, correlations and reliability coefficients [6], this is not always the case and might be at the expense of validity [6,14,16,17,19]. Consideration of how neurological respondents interpret rating scale response categories is therefore warranted in order to provide an evidence base for their selection when developing and modifying patient-reported rating scales. Additionally, it is unclear whether rating scale response category interpretations differ between people with long-term illnesses and control subjects, since we have been unable to identify any controlled studies of this kind. This may be particularly relevant in chronic unpredictable neurological disorders, such as PD, that impacts a variety of functions.

The objective of this study was to investigate the interpretation of commonly used rating scale response categories and to identify distinct and roughly equally spaced response categories for patient-reported rating scales in PD and age-matched control subjects.

## Methods

Two samples were used: 51 consecutive Swedish speaking people with neurologist diagnosed PD [20] without clinically significant mental impairments (e.g. dementia, confusion) were recruited from a Swedish university hospital, and 36 age-matched controls without neurological disorders were recruited through snowball sampling. In addition to age, it was desired that controls should have approximately the same educational background as the PD sample.

Participants were interviewed regarding demographic characteristics and self-completed the physical functioning and mental health scales of the SF-36 [21,22]. People with PD were also assessed regarding Hoehn & Yahr stages of PD severity [23]. Participants were then presented with 21 rating scale response options representing ratings of frequency, intensity and level of agreement (see Table 1). Respondents indicated their interpretation of each of the 21 response categories on 100-mm visual analog scales (VAS) anchored by *Never *- *Always *(frequency), *Not at all *- *Extremely *(intensity), and *Totally disagree *- *Completely agree *(agreement) [9,10]. Categories and anchors were taken from patient-reported rating scales used in PD [22,24-29]. Response categories were presented one at a time, on separate sheets and in random order; each sheet consisted of one response category and a corresponding 100-mm anchored VAS line. Before commencing this part of the data collection, the investigators ascertained that participants understood the task by explaining the procedure and its objective. In doing so, the task was illustrated by an example relating the word "warm" to a VAS line anchored by "ice cold" and "boiling hot". During data collection, any comments regarding the response categories and their interpretation were recorded. If a respondent was unable to assess the magnitude of a response category, this was recorded as a missing value.

**Table 1:** Descriptive response category VAS data

		Mean	**SD**	**95% CI**	**Median**	**q1-q3**	**Min-max**
Frequency:							
f1	Seldom (*sällan*)	25.3	16.4	21.8-28.8	20	14-31	3-72
f2	Occasionally (*vid enstaka tillfällen*)	30.8	20.5	26.4-35.2	24	15-40	4-82
f3	A little of the time (*lite av tiden*)	33.7	18.0	29.9-37.6	28	21-46	7-75
f4	Some of the time (*en del av tiden*)	44.7	16.8	41.1-48.3	43.5	33-54.5	15-81
f5	Sometimes (*ibland*)	45.9	17.5	42.2-49.6	48	33-52	14-91
f6	A good bit of the time (*en hel del av tiden*)	71.1	18.3	67.2-75	75	67-82	5-98
f7	Often (*ofta*)	74.7	12.7	72.0-77.4	76	70-83	32-98
f8	Most of the time (*största delen av tiden*)	76.8	16.3	73.3-80.3	80	71-89	21-99
							
Intensity:							
i1	A little bit (*en aning*)	21.9	15.2	18.6-25.1	19	11-28	2-76
i2	Slightly (*lite*)	24.0	14.1	21.0-27	21	14-27	2-78
i3	Somewhat (*något*)	29.8	17.4	26.1-33.6	26	17-37.5	2-83
i4	Moderately (*måttligt*)	42.1	14.3	39-45	44	31.8-50.2	6-86
i5	Quite a bit (*ganska mycket*)	73.5	11.9	71-76.1	74	66-81	38-96
i6	A lot (*mycket*)	73.7	14.1	70.7-76.6	76	69-82	24-99
							
Agreement:							
a1	Disagree (*stämmer inte*)	15.5	21.2	11-20	7	2-19	0-81
a2	Mostly false (*stämmer inte särskilt bra*)	28.0	17.7	24.2-31.8	23	16-34	4-84
a3	Don't know (*osäker*)	38.9	19.1	34.8-43	40	24-50	2-84
a4	Do not agree or disagree (*varken stämmer eller stämmer inte*)	43.2	16.0	39.8-46.6	49	41-52	0-71
a5	Mostly true (*stämmer ganska bra*)	70.7	14.7	67.6-73.8	74	63-81	32-94
a6	Agree (*stämmer*)	78.3	21.9	73.6-83	86	64-95	13-100
a7	Strongly agree (*stämmer helt*)	86.7	18.2	82.7-90.6	95	82.5-99	22-100

Visual analog scale (VAS) values for the 21 rating scale response categories as determined by people with Parkinson's disease and control subjects. Categories are organized in ascending order (from lower to higher mean VAS values). Swedish category wordings used in this study are given in parentheses ^a^

^a ^There were two instances of missing VAS data among people with Parkinson's disease (both involving category a7) and six missing VAS values among controls, including one each for categories i3 and i4, and three missing values for category a3.

SD, standard deviation; CI, confidence interval; q1-q3, 25^th ^and 75^th ^percentiles.

The study was reviewed by the institutional ethics advisory committee and was conducted in accordance with the Declaration of Helsinki. All participants provided written informed consent.

### Analyses

Statistical analyses were conducted using SPSS 14 for Windows (SPSS Inc., Chicago IL). P-values were adjusted for multiple testing using the Benjamini-Hochberg procedure [30], and considered statistically significant when < 0.05. The distribution of data was assessed regarding univariate and multivariate normality (Kolmogorov-Smirnov and Mardia's tests) and described and analyzed accordingly.

### Group comparisons of rating scale response category interpretations

Nonparametric multivariate analysis of variance (MANOVA) [31] was used to compare VAS values from PD and control respondents. If no significant differences between groups were identified, the pooled data was used to explore (using nonparametric MANOVA) whether VAS values differed according to educational level (university/professional degree vs others), age, physical functioning and mental health (with the latter three dichotomized by their median values). For the PD group, differences in VAS values according to PD duration (dichotomized by the median) and whether patients experienced dyskinesias or not were also explored.

### Identification of distinct rating scale response categories

To determine the best response options for the three types of ratings, the mean, standard deviations (SD) and 95% confidence intervals (CIs) of the VAS values were examined [9,10]. The criterion was that mean VAS values (or their associated 95% CIs) should be distributed equally across the 0-100 mm continuum, assuming the values of 0 and 100 for the predefined extreme anchor categories. This was done for three-, four-, five- and six-category response scales. For example, for a five-category response scale, the three response categories with mean VAS values closest to 25, 50 and 75 mm were identified and each 95% CI was examined to determine if it covered the criterion value. For three-, four- and six-category response scales the corresponding reference locations were 50 mm (three categories), 33 and 67 mm (four categories) and 20, 40, 60 and 80 mm (six categories). In addition to roughly equal distances between mean locations, the 95% CIs for the VAS values of the selected response categories should not overlap. If two or more response categories met these criteria, the one with the smallest dispersion (SD) was selected. Finally, participants' comments were also taken into account when determining response category suitability.

## Results

Sample characteristics are reported in Table 2. There were no differences between people with PD and controls regarding age, educational levels or mental health scores, but there were more men in the PD sample, and controls had better physical functioning scores than people with PD (Table 2). Data collection also took significantly longer for people with PD (mean [SD], 17.4 [7.2] minutes) than for controls (mean [SD], 12.7 [4.3] minutes) to complete (P = 0.001; unpaired t-test). In the PD group, 94% were treated with levodopa, 82% were on dopamine agonists, COMT- and MAO-inhibitors were used among 43% each, and 25% had undergone a neurosurgical intervention for their PD.

**Table 2:** Sample characteristics

	PD (n = 51)	Controls (n = 36)	**P-value **^**e**^
Male gender, n (%)	36 (71)	14 (39)	0.008 ^f^
Age, mean (SD)	66 (8.1)	66.2 (9.3)	0.889 ^g^
Academic/professional degree, n (%)	26 (51)	19 (53)	0.889 ^f^
Physical functioning, median (q1-q3) ^a^	75 (50-90)	90 (80-95)	0.008 ^h^
Mental health, median (q1-q3) ^a^	76 (64-88)	84 (76-92)	0.068 ^h^
PD duration (years), mean (SD)	9.8 (5.6)	-	-
Hoehn & Yahr ("on"), median (q1-q3; min-max) ^b,c^	II (II-III; I-V)	-	-
Hoehn & Yahr ("off"), median (q1-q3; min-max) ^b,d^	III (III-IV; I-V)	-	-
Motor fluctuations, n (%)	36 (71)	-	-
Dyskiesias, n (%)	25 (49)	-	-

^a ^According to the Physical Functioning and Mental Health scales of the SF-36. Possible score range, 0-100 (100 = better).

^b ^Range, I-V (I = mild unilateral disease; II = Bilateral disease without postural impairment; III = Bilateral disease with postural impairment, moderate disability; IV = Severe disability, still able to walk and stand unassisted; V = Confined to bed or wheelchair unless aided) [23].

^c ^As determined for the "on" phase, i.e. periods with good anti-parkinsonian drug response.

^d ^As determined for the "off" phase, i.e. periods with poor or no anti-parkinsonian drug response.

^e ^Adjusted for multiple testing using the Benjamini-Hochberg procedure [30].

^f ^Chi-square test.

^g ^Independent samples t-test.

^h ^Mann-Whitney U-test.

PD, Parkinson's disease; SD, standard deviation; q1-q3, 25^th ^and 75^th ^percentiles.

## Group comparisons of rating scale response category interpretations

MANOVA of overall differences among VAS values between PD and control groups was not significant (P = 0.286). Similarly, MANOVAs of VAS values for the pooled sample did not display any significant differences between educational levels (P = 0.220), age groups (P = 0.220), or between people with lower and higher physical functioning (P = 0.501) and mental health (P = 0.238) scores. In the PD sample, there were no differences between people with shorter and longer disease durations (P = 0.213) or between those with or without dyskinesias (P = 0.212).

## Identification of distinct rating scale response categories

**Figure 1:**
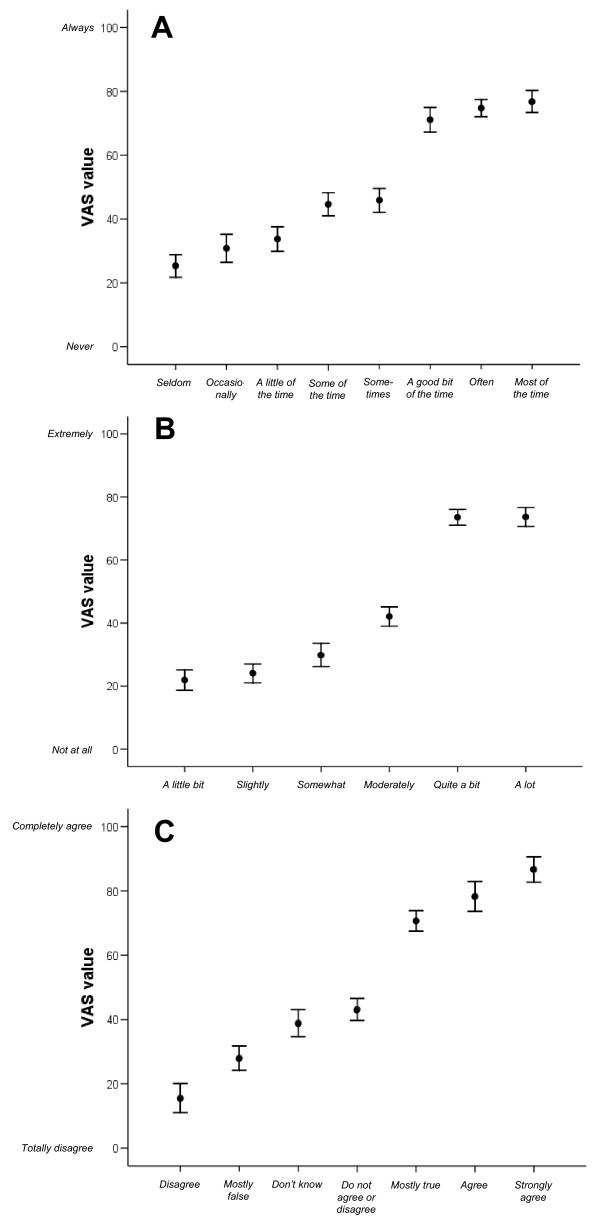
**Response category mean VAS values and 95% CIs**. Mean values (black dots) with associated 95% confidence intervals (error bars) for 100-mm visual analog scale (VAS) ratings (y-axis) of the perceived meaning of response category wordings (x-axis) in relation to (A) *Never *(0 mm) - *Always *(100 mm), (B) *Not at all *(0 mm) - *Extremely *(100 mm), and (C) *Totally disagree *(0 mm) - *Completely agree *(100 mm) among people with Parkinson's disease (n = 51) and an age-matched control group (n = 36). See Methods for details.

Results from the VAS evaluations of the 21 rating scale response categories from the pooled sample are presented in Table 1 and Figure 1, with categories organized in ascending order (from lower to higher mean VAS values) within each of the three response category types. Additional file 1 presents the corresponding data separately for people with PD and controls.

One third (n = 12) of the control group and 43% (n = 22) of the PD group expressed difficulties interpreting the response category *Don't know*. Difficulties were also expressed by one or two respondents each for the categories *Sometimes*, *Somewhat*, *Moderately*, and *Do not agree or disagree*.

**Figure 2:**
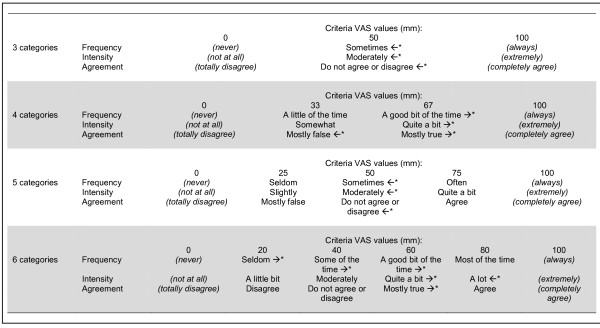
**Selected response categories**. Selected response categories for three-, four-, five-, and six-category response scales as determined from observed visual analog scales (VAS) values. See Methods for details. * 95% CI of category VAS value does not cover criterion value. Arrows indicate direction of discrepancy (←, below criterion value; →, above criterion value); see Table 1 for raw data.

Based on these observations the best three-, four-, five and six-category response scales according to the pre-defined criteria are provided in Figure 2. It can be seen that the equal distances criterion was not fully met in either of the identified three-, four-, five or six-category response scales for any of the three types of ratings. The proportion of categories whose 95% CI covered the criterion VAS values was highest for the six-category agreement scale (75%), followed by the five category scales for all three types of ratings (67%).

## Discussion

This appears to be the first controlled study on the interpretation of patient-reported rating scale response categories in the clinical neurosciences. As such, it provides a first evidence base and initial guidance for selection of rating scale response categories when developing new or modifying available patient-reported rating scales for PD. This is highly relevant as clarity, distinctiveness and equality of response category intervals represent fundamental assumptions underpinning traditional rating scale construction [1,32] that are recognized by, e.g. the FDA when judging the appropriateness of rating scales as clinical trial endpoints [5]. Although focusing on PD, the lack of systematic differences between people with PD and age-matched controls, as well as between other health-related respondent characteristics, suggests that our findings are relevant beyond this context.

The identified best categories for three-, four-, five and six-category response scales were not optimal, as they failed to fulfill the assumption of equal inter-category distances also when considering their 95% CIs. For example, the distances between *Some of the time *and *A good bit of the time *are clearly different from those between *A good bit of the time *and *Most of the time*. Extrapolating data from this study to response categories in commonly used scales reveals similar problems. For example, the three non-extreme response options in the original PDQ-39 (*Occasionally *- *Sometimes *- *Often*) [27] correspond to mean VAS locations of 30.8, 45.9 and 74.7, respectively. That is, the estimated distance between the latter two categories is about twice as large as that between the former two. Similar or more extreme situations are evident with scales such as the PFS-16 [24], FACIT-F [29], SF-36 [22], PDQL [25], and PDQUALIF [28].

Conceivably, this has at least two consequences. First, it may contribute to respondent difficulties in using the response options. Second, it is unknown what a certain difference in raw rating scale scores represents and by how much more someone has changed compared to people with smaller change scores. This illustrates the ordinal nature of raw rating scale data and argues against the legitimacy of analyzing and interpreting summed integral numerals from item responses as linear measures [3,33,34]. This latter aspect represents a fact perhaps partly overlooked when developing rating scales; that is, the profound step that is taken when transforming words (qualitative descriptors) into numbers (quantities) that typically are treated as linear measures.

There are a number of aspects that need to be taken into consideration when interpreting the results presented here. First, the appropriateness of using VAS to evaluate participants' interpretation of response categories may be questioned since evidence speaks against the linearity of VAS data [35]. However, there is also evidence supporting the linearity of VAS ratings [36,37], and the approach has been found useful in previous studies of rating scale category interpretations [9-11]. Second, our observations refer to the Swedish versions of the studied response categories, and the equivalence between various language versions is dependent on cultural and semantic aspects, as well as the quality of the translation. It has for example been shown that interpretations of the same response category can differ between languages as well as between cultures within the same language [11]. However, the VAS values found here are in general agreement with those reported in previous studies using the same methodology and response categories [9,10]. This suggests that our observations are not necessarily limited to a Swedish context. Third, we limited the types of response categories to frequency, intensity and agreement, and there are also response categories of these types that were not covered here. Furthermore, the anchor categories were assumed to have fixed values at 0 and 100 mm, whereas their interpretations actually may differ between people. For example, studies investigating the perceived absolute frequency or probability of occurrence associated with frequency descriptors have found variations in the interpretation of *Always *as well as *Never *[38,39].

The samples studied here were not randomly selected, which may limit the generalizability of results. Furthermore, the sample sizes were somewhat limited, which influences the precision of observations and, therefore, renders the reported 95% CIs wider than otherwise would have been the case. However, given that data failed to support the assumption of equal inter-category distances even with consideration of the observed CIs, increasing the number of observations would presumably have yielded even stronger evidence against legitimate raw score summation of the response categories studied here. Similarly, the lack of differences between people with PD and control subjects, as well as between other subgroups also needs to be interpreted in view of the sample size. That is, with increasing numbers of observations, statistically significant differences are increasingly likely to be detected. However, statistical significance says nothing about the practical significance of differences, which is not known for the current type of data.

The variability in interpretations of response categories was wide between individuals (as illustrated by the ranges of VAS values). This does not appear to be limited to patient-reported data, as studies regarding physicians' interpretation of various probability related expressions (including some of the response categories studied here) have shown similar variability [38]. This variability further complicates score interpretation at the individual patient level. An important aspect in this respect is the extent to which interpretations are stable within individuals over time. This needs to be assessed in further studies designed for this purpose. Such studies would also allow for direct evaluation of the error variation in VAS ratings, which is an important aspect for the interpretability of data that was not considered in this study.

Our observations concern the interpretation of response categories without reference to a particular context. This is different from the use of response categories in rating scales where items articulate the context within which responses are requested. Studies have shown that the meaning of descriptors of, e.g. frequency differ according to context as well as respondents' experiences within the context [32,40]. While this hampers the possibilities to make valid comparisons of raw rating scale data between people and between scales tapping different variables, the magnitude of these effects for various health outcome variables is uncertain and will need to be addressed in future studies.

A large proportion of respondents expressed difficulties with the response category *Don't know*. This observation is in accordance with previous studies of neutral middle categories (e.g., *Undecided*, *?*, and *Not sure*) in Likert type response scales [19,41,42]. These studies have shown that there may be a variety of reasons why respondents select this type of response category and that in practice, it does not operate as a middle category. It has therefore been recommended that it should not be presented as an integral part of a continuum of levels of agreement but, if used at all, be presented separately from categories expressing agreement levels [41]. The observations reported here provide further qualitative evidence in support for this notion.

The ordinal nature of rating scale response categories challenges the legitimacy of summing individual item scores into total scores, as well as their interpretability [3,4,34]. However, there are means to empirically determine how the response categories used with a particular set of items function when administered to a particular group of people, and to overcome the assumption of equal intervals in the construction of total scores. Specifically, the polytomous Rasch measurement model for ordered response categories does not assume equal intervals between response categories, tests whether thresholds between adjacent categories are ordered in the expected manner, and provides a means of exploring the effect of collapsing adjacent categories [19,41,43,44]. Additionally, the Rasch model defines, mathematically, the requirements that data need to meet in order to produce measurements, and when these requirements are met scores can be expressed as invariant measures instead of ordinal numbers [33,45-47]. This study argues for a wider application of this methodology, including appropriate appreciation of response category functioning, whenever rating scale data are used for measurement. For purposes of assessment (in contrast to measurement [33,46,48]) an alternative to summed total scores that takes the ordinal nature of rating scale response categories into consideration would, e.g., be the approaches proposed by Svensson [49].

## Conclusions

Although in need of replication and extension, this study offers an evidence base for selecting more interpretable patient-reported rating scale response categories. As such, it provides guidance when developing new or modifying existing rating scales. However, it must be stressed that the selection of response categories also should be guided by additional considerations, so that they express levels of the construct articulated by the items in a meaningful way that is congruent with the intention of the scale. In this perspective, response categories alternative to those primarily identified here may be appropriate, particularly since the difference between identified and alternative categories in some cases were marginal. Our observations also illustrate problems associated with raw rating scale data that clinicians and investigators need to be aware of and that argue for the application of newer rating scale methodologies such as Rasch measurement. Response categories need to be treated with rigour in the construction and application of rating scales.

## Abbreviations

CI: Confidence interval; COMT: Catechol-O-methyl transferase; FACIT-F: Functional Assessment of Chronic Illness Therapy - Fatigue; FDA: Food and Drug Administration; MANOVA: Multivariate analysis of variance; MAO: Monoamine oxidase; mm: Millimeter; PD: Parkinson's disease; PDQ-39: 39-item Parkinson's disease questionnaire; PDQUALIF: Parkinson's disease quality of life scale; PFS-16: 16-item Parkinson fatigue scale; SD: Standard deviation; SF-36: Medical Outcomes Study 36-item Short Form health survey; VAS: Visual analog scale;

## Competing interests

The authors declare that they have no competing interests.

## Authors' contributions

IK participated in designing the study, data collection, data analyses and interpretation, and drafting of the manuscript. HR participated in designing the study, data collection, data analyses and interpretation. JR participated in data collection, and drafting of the manuscript. PN participated in designing the study, conducted data analyses, participated in data interpretation, and drafting of the manuscript. PH conceptualized and designed the study, participated in data collection, conducted data analyses, participated in data interpretation and drafted the manuscript. All authors read and approved the final manuscript.

## Supplementary Material

Additional file 1 **Descriptive response category VAS data separately for people with Parkinson's disease and control subjects**. Click here for file
