# Supplementary material for: Interpretation of response categories in patient-reported rating scales: a controlled study among people with Parkinson's disease
Source: Health Qual Life Outcomes. 2010 Jun 24;8:61. doi: 10.1186/1477-7525-8-61 (PMC2908570; doi:10.1186/1477-7525-8-61)
Supplement: Additional file 1 — Descriptive response category VAS data separately for people with Parkinson's disease and control subjects. [file 1477-7525-8-61-S1.PDF]

Additional file 1 Visual analog scale (VAS) values for the 21 rating scale response categories as determined by people with Parkinson's disease and control subjects. Categories are organized in ascending order (from lower to higher mean VAS values) according to the values from the Parkinson's disease sample. Swedish category wordings used in this study are given in parentheses.

	Parkinson's disease (n=51)						Controls (n=36)					
	Mean	SD	95% CI	Median	q1-q3	Min-max	Mean	SD	95% CI	Median	q1-q3	Min-max
Frequency:												
Seldom (*sällan*)	28.1	17.2	23.3-33.0	22	16-37	4-72	21.3	14.6	16.3-26.2	18.5	11-26	3-68
Occasionally (*vid enstaka tillfällen*)	34.4	21.1	28.4-40.3	29	19-48	6-82	25.7	18.9	19.3-32.1	20.5	14-31	4-81
A little of the time (*lite av tiden*)	37.9	19.4	32.4-43.4	32	22-56	9-75	27.8	14.4	23.0-32.7	24	20-32	7-72
Some of the time (*en del av tiden*)	46.2	17.7	41.1-51.2	47	32-58	15-81	42.6	15.5	37.4-47.8	38	33-51.5	16-77
Sometimes (*ibland*)	49.1	15.0	44.8-53.3	49	38-54	14-78	41.4	19.9	34.7-48.2	38	25-52	16-91
A good bit of the time (*en hel del av tiden*)	71.6	15.7	67.2-76.0	74	69-82	19-95	70.4	21.8	63.0-77.8	77.5	63.5-82	5-98
Most of the time (*största delen av tiden*)	74.2	16.4	69.6-78.8	77	69-87	21-93	80.5	15.6	75.2-85.8	84.5	75-90	30-99
Often (*ofta*)	74.4	13.1	70.7-78.1	76	70-82	32-98	75.1	12.3	71-79.3	75.5	69-85	46-94
Intensity:												
A little bit (*en aning*)	24.6	16.8	19.8-29.3	20	13-32	4-76	18.1	11.7	14.1-22.0	16	10-24	2-60
Slightly (*lite*)	25.8	15.2	21.5-30.0	23	16-28	2-78	21.6	12.0	17.6-25.7	18.5	12.5-25	6-48
Somewhat (*något*)	33.0	18.8	27.7-38.3	26	19-42	9-83	25.2	14.1	20.4-30.1	21	15-37	2-58
Moderately (*måttligt*)	42.7	16.2	38.2-47.3	44	31-52	12-86	41.1	11.0	37.4-45.0	45	34-50	6-53
A lot (*mycket*)	69.8	13.4	66.0-73.6	73	63-79	30-93	79.1	13.2	74.6-83.6	79	75-87.5	24-99
Quite a bit (*ganska mycket*)	73.1	10.7	70.1-76.1	74	66-79	44-96	74.1	13.6	69.5-78.7	75.5	67-85.5	38-92
Agreement:												
Disagree (*stämmer inte*)	20.7	24.3	13.9-27.6	13	4-25	0-81	8.1	12.7	3.8-12.4	4.5	1-8	0-63
Mostly false (*stämmer inte särskilt bra*)	31.2	19.1	25.8-36.6	27	18-37	6-84	23.4	14.7	18.4-28.4	20.5	15-30	4-67
Do not agree or disagree (*varken stämmer eller stämmer inte*)	41.2	17.5	36.3-46.2	47	35-51	0-71	45.9	13.3	41.4-50.4	50	45.5-52	1-58
Don't know (*osäker*)	42.1	20.7	36.3-47.9	46	27-55	2-84	33.9	15.3	28.5-39.4	33	21-47.5	7-61
Mostly true (*stämmer ganska bra*)	70.2	15.3	65.9-74.5	75	64-80	32-93	71.4	14.1	66.6-76.2	73.5	62-81	32-94
Agree (*stämmer*)	75.6	21.9	69.4-81.8	82	56-95	21-100	82.1	21.6	74.8-89.4	92.5	74-98	13-100
Strongly agree (*stämmer helt*)	83.3	20.1	77.5-89.0	90	75-98	22-100	91.3	14.3	86.5-96.1	97.5	89-100	36-100

SD, standard deviation; CI, confidence interval; q1-q3, 25th and 75th percentiles.
